# Public Health Approach to Developing Palliative Care

**DOI:** 10.31729/jnma.7255

**Published:** 2022-08-31

**Authors:** Kedar Prasad Baral, Kiweta Bista, Chiniya Lama, Paras Kumar Acharya, Pradeep Vaidya, Rajesh Nath Gongal

**Affiliations:** 1 School of Public Health and Department of Community Health Sciences, Patan Academy of Health Sciences, Lagankhel, Lalitpur, Nepal; 2 Community Based Palliative Care Programme, Patan Academy of Health Sciences, Lagankhel, Lalitpur, Nepal; 3 Department of Internal Medicine, Patan Academy of Health Sciences, Lagankhel, Lalitpur, Nepal; 4 Department of Surgery, Tribhuvan University Teaching Hospital, Maharajgunj, Kathmandu, Nepal; 5 Department of Surgery, Patan Academy of Health Sciences, Lagankhel, Lalitpur, Nepal

**Keywords:** *community health care*, *Nepal*, *palliative care*, *public health*

## Abstract

Nepal has witnessed demographic and epidemiological transition resulting in the shift from infectious diseases to non-communicable diseases as the major disease burden. Around 60% of mortalities and morbidities are attributable to non-communicable diseases of which the majority end with the need for palliative care services. The current palliative care services in Nepal are in the infancy stage compared with other services. Undignified dying is a challenging public health problem and as such requires a public health approach to address it with the involvement of all stakeholders. Recognizing the need for the end spectrum of non-communicable diseases patients, the Ministry of Health, Nepal recently introduced the policy to address the unmet need through the community-based palliative care program, a laudable initiation.

## INTRODUCTION

Palliative care is an approach that improves the quality of life of patients and families facing the problem associated with a life-threatening illness. The demographic and epidemiological transition resulted in a shift from infectious to non-communicable diseases.^1^ Over 60% of morbidities and mortalities are attributable to non-communicable diseases (NCDs), injuries, and nutritional conditions.^2^ Majority of these end in need of palliative care services. However, only a few are able to access it.

## NEED VERSUS ACCESS

A study estimated that there are 52,000 patients in Nepal requiring palliative care every year, which was considered an underestimate.^3^ A study in the rural community of Makawanpur showed that 6.4% of the population had NCDs and 3.04% required palliative care using the Supportive and Palliative Care Indicators Tool (SPICT) criteria.^4^ Although there has been some progress in the field of palliative care from when Hospice Nepal, the first organisation to start palliative care in Nepal, was established in the year 2000, only a fraction of people in need actually receive palliative care; many die without any support.^5^ It can be safely assumed that "dying poorly" or " bad death" in Nepal is a public health problem of immense proportion. A public health problem requires a public health approach. World Health Organization (WHO) has also recommended public health approach to developing palliative care with its four pillars, namely appropriate policy, adequate drug availability, education of policymakers, health workers, and the public, and implementation of palliative care services at all levels throughout the society.^6^

In the public health history of Nepal, palliative services have not come as part of the continuum of integrated care packaging in the national policy. Until recently, Nepal's focus was on infectious diseases and was limited to acute care management. Palliative services were the least discussed among health professionals and academia. Current service availability is limited to a few places; sporadic, isolated, and uncoordinated.

A few attempts from the Nepalese Association of Palliative Care, Hospice Nepal, and the community-based model in Thaha Municipality were noted. Still, these are not enough to create a public health impact.

## DRAWING LESSONS FROM PAST

The achievement of Nepal's health program has been based on the primary health care approach up to the community level through community-based programs. Despite the slow economic development, poor infrastructure, political instability, and ten years of violent conflict there has been an increase in life expectancy from 28 years in the 1950s to more than 71 currently.^2,7^

Over the last 70 years, Nepal introduced various public health programs addressing major health problems of the period to contribute to health, wellbeing, and development. All the past public health programs contributed to Nepal's rapid and steady health improvement and were among a few countries in the world meeting the Millennium Development Goals target.

Therefore, to develop palliative care in the country, we must draw lessons from our past achievements and adopt Nepal's rich learning and experiences over the last 6-7 decades. Engagement of the community by educating and empowering them and making them responsible and using the resources including human resources already in place as part of the health delivery system is key to the development of palliative care services in the country.

As enshrined in the Constitution of Nepal 2015, health is a fundamental right of all the citizens of Nepal. To realise these constitutional rights, the government of Nepal formulated and introduced National Health Policy 2019 and articulated the maintenance of the pace of current achievement through improving 'health systems as per the federal structure'. The most notable policy statement found in the context of public health prioritisation as stated in the policies 6.5 of National Health Policy 2019, "in accordance with the concept of universal coverage, promotional, preventive, curative, rehabilitative and palliative services shall be developed and expanded in an integrated manner".^8^

## WAY FORWARD

It is the right of every citizen not only to live well but also to die well. In this context, the current policy was found timely to address the epidemiological shift of the health problem of Nepal. An appropriate and laudable step came when the government of Nepal announced the implementation strategy through the Community Based Approach mentioned in point 35 of the Budget 2078-2079 to actualize the health policy.

These are all-welcoming steps, but there are challenges ahead. Nepal's health achievement is the result of a cost-effective community-based primary health care approach. The right to die well: how do we deliver this? How we design and implement a palliative care program will determine whether or not we will bring palliative care service delivery from the periphery to the centre of Nepal's public health system as a part of community-based primary health care within the national priority package.
